# Brachytherapy Combined With or Without Hormone Therapy for Localized Prostate Cancer: A Meta-Analysis and Systematic Review

**DOI:** 10.3389/fonc.2020.00169

**Published:** 2020-02-19

**Authors:** Xueliang Zhou, Dechao Jiao, Mengmeng Dou, Jianjian Chen, Bin Han, Zhaonan Li, Yahua Li, Juanfang Liu, Xinwei Han

**Affiliations:** ^1^Department of Interventional Radiology, The First Affiliated Hospital of Zhengzhou University, Zhengzhou, China; ^2^Department of Neurology, The First Affiliated Hospital of Zhengzhou University, Zhengzhou, China; ^3^Radiotherapy Department, The First Affiliated Hospital of Zhengzhou University, Zhengzhou, China

**Keywords:** prostate cancer, brachytherapy, hormone replacement therapy, survival, meta-analysis

## Abstract

**Purpose:** The purpose of this study was to evaluate the efficacy of brachytherapy combined with or without hormone therapy in patients with localized prostate cancer.

**Methods and Materials:** We systemically searched the Medline, Web of Science, Cochrane Library and Embase databases for studies published between the databases' dates of inception and February 2019. The primary endpoints were the 5-year overall survival (OS) rates, 5-year biochemical progression-free survival (bPFS) rates and 10-year bPFS rates. The results were expressed as the relative risk (RR) and 95% confidence interval (CI). Based on the heterogeneity evaluated with the *I*^2^ statistic, a meta-analysis was performed using either a random- or fixed-effects model.

**Results:** A total of 16 cohort studies including 9,359 patients met all the criteria for inclusion in the analysis. Our data showed that brachytherapy (BT) combined with hormone therapy (HT) increased the patients' 5-year bPFS rates (RR = 1.04, 95% CI: 1.01–1.08, *P* = 0.005) and 10-year bPFS rates (RR = 1.12, 95% CI: 1.02–1.23, *P* = 0.001) compared with BT monotherapy. However, BT combined with HT did not increase the patients' 5-year OS rates (RR = 1.02, 95% CI: 0.99–1.095, *P* = 0.1) compared with BT monotherapy.

**Conclusions:** BT combined with HT can increase the bPFS rates of patients with localized prostate cancer, but it does not improve patients' OS rates.

## Introduction

Prostate cancer is the fifth leading cause of cancer-related deaths worldwide, and it is also one of the most common malignant tumors among men in developed countries (1). Prostate cancer accounted for nearly one-fifth of all newly diagnosed cancers in the United States in 2018 (2). The main treatments for localized prostate cancer include radical prostatectomy (RP), external beam radiation therapy (EBRT) and brachytherapy (BT) of the prostate (3, 4). Patients with localized prostate cancer can choose from a variety of treatment options. A systematic review showed that EBRT, BT and RP were effective monotherapies for localized prostate cancer and that BT had a similar biochemical progression-free survival (bPFS) rate as RP in patients with a low to moderate risk of prostate cancer (5). The results of randomized clinical trials have shown that EBRT combined with BT, compared to EBRT monotherapy, improves the bPFS rates of patients with intermediate- and high-risk prostate cancer (6, 7).

However, a retrospective trial found that BT reduced biochemical failure in patients with predominantly intermediate- and high-risk disease compared with EBRT alone (8). Prostate BT not only has a better therapeutic effect but also obvious dosimetric advantages and lower costs compared with EBRT (9, 10). Hormone therapy (HT) has been shown to improve the prognoses of patients with locally advanced prostate cancer receiving EBRT (11). BT has become an increasingly popular treatment option for localized prostate cancer. Lee et al. believe that BT combined with HT can improve the bPFS rates of patients compared with BT monotherapy (12), whereas others argue that BT combined with HT does not improve the bPFS rates of patients compared with BT monotherapy (13, 14). However, there is a lack of systematic reviews on the application of BT combined with HT in patients with prostate cancer.

In this meta-analysis, we aimed to determine whether BT combined with HT could increase the bPFS and overall survival (OS) rates of patients with localized prostate cancer.

## Materials and Methods

The study protocol is registered through PROSPERO, and the registration number is CRD42019126003, which can be found online at https://www.crd.york.ac.uk/PROSPERO/display_record.php?RecordID=126003.

### Search Strategy

This meta-analysis followed the Preferred Reporting Items for Systematic Reviews and Meta-Analyses (PRISMA) criteria (15). A comprehensive search was conducted using the Medline (www.ncbi.nlm.nih.gov/pubmed), Web of Science (http://apps.webofknowledge.com), Cochrane Library (www.cochranelibrary.com) and Embase (www.embase.com) databases for English language studies published between the databases' dates of inception and February 2019. The searches involved a combination of medical-subject heading searches and text words. We searched these databases using the following terms: “prostate cancer,” “prostate carcinoma,” “prostatic neoplasms,” “brachytherapy,” “hormone,” and “androgen.” The detailed search strategy is shown in Supplementary Table S1.

### Inclusion and Exclusion Criteria

The following inclusion criteria were developed to guide the selection of studies: (1) a clinical trial or a prospective or retrospective study; (2) a study design with an observation group that received a combination of BT combined with HT and a control group that received BT monotherapy; (3) original full-text articles designed to evaluate the association between a therapy and the bPFS or OS of patients with prostate cancer; and (4) studies utilizing appropriate statistical methods for analyses and having sufficient data.

The following studies were excluded from the meta-analysis: (1) reviews, letters, case reports, conference papers, and studies based on animal models or cell models; (2) duplicated studies; and (3) studies lacking sufficient data for extraction.

### Study Selection and Data Extraction

Two reviewers independently screened titles and abstracts based on the above criteria and evaluated the eligibility of each study by reading the full text. Two researchers independently extracted data from the articles. Disagreements were resolved by consensus following a literature reanalysis. We extracted the following information from each included article: (1) general study information, including the first author, date of publication, country, and study design; (2) patient characteristics, including the number of participants, age and risk group; (3) treatment outcomes, including 5-year OS, 5-year bPFS, and 10-year bPFS; and (4) the methodological quality assessment index.

### Quality Assessment

The Newcastle-Ottawa Scale (NOS) was used to evaluate the quality of the included publications, as all of them were retrospective cohort studies (16). The assessment of quality using the NOS is based on three parameters: selection, comparability and outcomes. Studies can receive a maximum possible score of nine stars. NOS scores of 7–9 indicate high-quality reports, and scores of 4–6 represent medium-quality reports.

### Statistical Analyses

The clinical outcomes of interest included 5-year OS and 5-year and 10-year bPFS. All statistical analyses were performed using STATA 14.0 (College Station, Texas 77845, USA, Serial number: 401406267051). Relative risk (RR), as the effect size for OS or bPFS, was expressed along with the 95% confidence interval (CI). RRs >1 and 95% CIs that did not overlap with 1 were indicators that BT combined with HT increased the length of OS or bPFS of patients with prostate cancer, whereas RRs <1 were indicators that BT combined with HT did not increase the length of OS or bPFS of patients with prostate cancer. The heterogeneity was evaluated using the Higgins *I*^2^ test and Cochran's Q test, with a significance level of *I*^2^ > 50% or *p* > 0.1. Fixed-effect models were used for the initial analyses after random-effect models were performed for validation analyses if significant heterogeneity was present. Publication bias was assessed through Begg's funnel plot (17) and Egger's linear regression (18). If the two-tailed *P*-value yielded by Egger's test was <0.1, Duval and Tweedie's trim and fill method for bias correction was used (19).

## Results

### Study Selection and Characteristics

**Figure 1:**
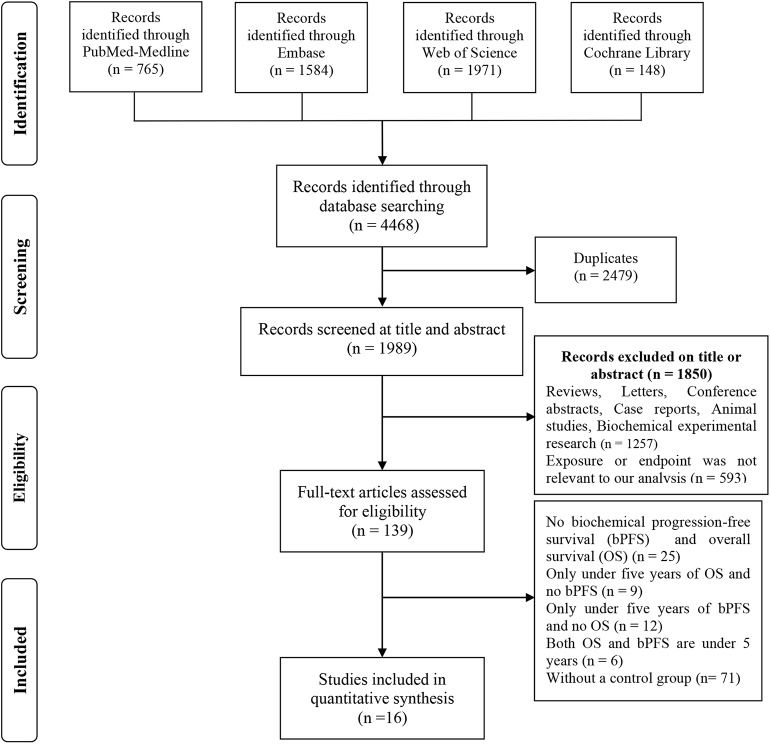
Flow diagram of the process by which the studies included in the analysis were selected.

We initially identified 558 potentially eligible studies using search terms. In the first screening, duplicate studies, reviews, letters, case reports, conference papers and studies based on animal models or cell models were excluded. The titles and abstracts of the remaining 139 studies were carefully reviewed, and 110 studies with irrelevant subjects were excluded. Two studies were excluded because they were published by the same institution. A full-text review of the remaining 27 studies was conducted to exclude studies that did not have survival data or did not fully meet the inclusion criteria. Finally, 16 studies with 9,359 patients (12–14, 20–32) were ultimately included in this study. A detailed outline of the process of study selection is presented in Figure 1.

All 16 studies were retrospective in nature; five were published in Europe (Germany, the UK and Italy), and 11 were published in North America (Canada and the USA). A total of 16 studies with 9,359 patients were analyzed, including 4,175 who were treated with BT combined with HT and 5,184 treated with HT monotherapy. Of all the studies, three reported 5-year OS rates of patients with prostate cancer, 12 reported 5-year bPFS rates, and four reported 10-year bPFS rates. The characteristics and clinical results of the included studies are summarized in Table 1. According to the NOS, the quality of the 16 studies was high, and the individual ratings for each study are presented in Table 2.

**Table 1:** Characteristics of the studies included in the meta-analysis.

**Study (year)**	**Study design**	**Country**	**Median age (year)**	**Treatment method**		**Number of patients (*****n*****)**		**Risk group (*****n*****)**			**5-year OS rate**		**5-year bPFS rate**		**10-year bPFS rate**	
				**EG**	**CG**	**EG**	**CG**	**Low**	**Intermediate**	**High**	**EG**	**CG**	**EG**	**CG**	**EG**	**CG**
Zimmermann et al. (20)	RCS	Germany	NA	BT + HT	BT	205	705	316	572	22	NA	NA	94.6%	94.6%	NA	NA
Pickles et al. (13)	RCS	Canada	69	BT + HT	BT	121	139	NA	NA	NA	NA	NA	86%	85%	NA	NA
Boehm et al. (21)	RCS	Europe	69	BT + HT	BT	213	206	67	276	76	93%	92%	86%	74%	NA	NA
Strom et al. (22)	RCS	America	65	BT + HT	BT	55	65	0	92	28	100%	98%	94.5%	93.8%	NA	NA
Dickinson et al. (23)	RCS	UK	62	BT + HT	BT	231	807	1038	0	0	NA	NA	96.8%	93.4%	NA	NA
Guarneri et al. (24)	RCS	Italy	68.3	BT + HT	BT	52	115	103	53	NA	NA	NA	79.1%	89%	NA	NA
Yamoah et al. (25)	RCS	America	NA	BT + HT	BT	525	863	963	369	56	NA	NA	NA	NA	89.3%	83.4%
Kubicek et al. (26)	RCS	America	70	BT + HT	BT	588	236	329	300	195	NA	NA	81.6%	80.1%	45.4%	35.9%
Peters et al. (27)	RCS	America	67	BT + HT	BT	975	762	761	400	576	NA	NA	95.6%	88.8%	NA	NA
D'Amico et al. (28)	RCS	America	72.7	BT + HT	BT	254	221	0	0	475	96.9%	93.2%	NA	NA	NA	NA
Merrick et al. (29)	RCS	America	68	BT + HT	BT	119	85	0	0	204	NA	NA	NA	NA	91.6%	79.7%
Stone et al. (30)	RCS	America	67	BT + HT	BT	64	215	146	0	133	NA	NA	NA	NA	94%	75%
Ash et al. (31)	RCS	UK	63	BT + HT	BT	346	321	NA	NA	NA	NA	NA	76.1%	72.6%	NA	NA
Lee et al. (12)	RCS	America	68	BT + HT	BT	133	68	0	66	135	NA	NA	79%	54%	NA	NA
Merrick et al. (32)	RCS	America	68	BT + HT	BT	162	245	157	162	88	NA	NA	99.4%	92.2%	NA	NA
Potters et al. (33)	RCS	America	NA	BT + HT	BT	132	131	NA	NA	NA	NA	NA	87.1%	86.9%	NA	NA

*RCS, Retrospective Cohort Study; EG, Experimental Group; CG, Control Group; BT, brachytherapy; HT, hormone therapy; OS, Overall survival; Bpfs, Biochemical progression-free survival; NA, Not Available*.

**Table 2:** The Newcastle-Ottawa Scale (NOS) for assessing the quality of the cohort studies.

**Studies**	**Selection**			**Comparability**			**Assessment of outcome**			**Total score**
**References**	**Representativeness of Exposure arm(s)**	**Selection of the comparative arm (s)**	**Origin of exposure source**	**Demonstration that outcome of interest was not present at start of study**	**Studies controlling the most important factors**	**Studies controlling the other main factors**	**Assessment of outcome with independency**	**Adequacy of Follow up length (to assess outcome)**	**Lost to follow up acceptable (less than 10% and reported)**	
Zimmermann et al. (20)	*	*		*	*		*	*	*	7
Pickles et al. (13)	*	*		*	*		*	*	*	7
Boehm et al. (21)	*	*	*	*	*		*	*	*	8
Strom et al. (22)	*	*		*	*		*	*	*	7
Dickinson et al. (23)	*	*	*	*	*		*	*	*	8
Guarneri et al. (24)	*	*	*	*	*		*	*	*	8
Yamoah et al. (25)	*	*	*	*	*		*	*	*	8
Kubicek et al. (26)	*	*	*	*	*		*	*	*	8
Peters et al. (27)	*	*	*	*	*		*	*	*	8
D'Amico et al. (28)	*	*	*	*	*	*	*	*	*	9
Merrick et al. (29)	*	*	*	*	*		*	*	*	8
Stone et al. (30)	*	*		*	*		*	*	*	7
Ash et al. (31)	*	*		*	*		*	*	*	7
Lee et al. (12)	*	*	*	*	*	*	*	*	*	9
Merrick et al. (32)	*	*	*	*	*		*	*	*	8
Potters et al. (33)	*	*	*	*	*		*	*	*	8

### Biochemical Progression-Free Survival

**Figure 2:**
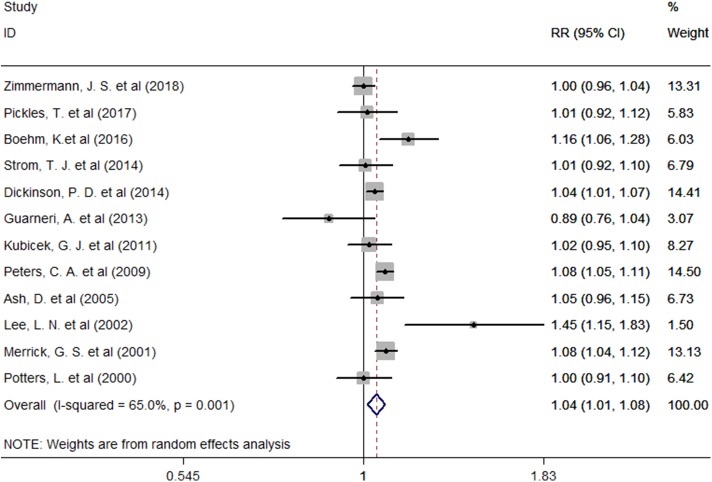
Forest plots of the association between BT combined with HT and the 5-year bPFS rates in patients with prostate cancer. RR, Relative Risk; CI, Confidence Interval.

**Figure 3:**
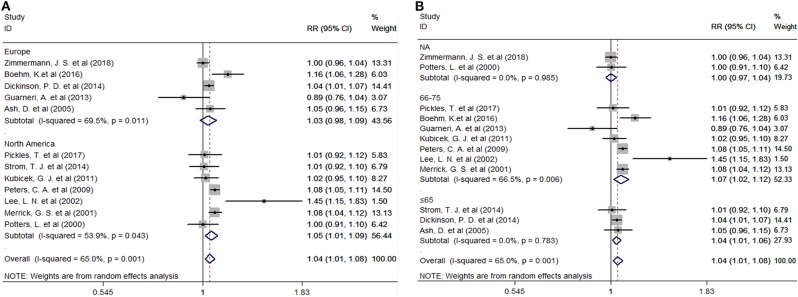
**(A)** Subgroup analyses by region. **(B)** Subgroup analyses by median age. RR, Relative Risk; CI, Confidence Interval.

Twelve studies provided the number of patients in the experimental and control groups and their 5-year bPFS rates. A forest plot of the association between BH combined with HT and the 5-year bPFS rates of patients with prostate cancer is shown in Figure 2. However, our analysis uncovered evidence of the presence of heterogeneity among the studies (Q = 31.4, *I*^2^ = 65.0%, *P* = 0.001, Tau^2^ = 0.0014). The results of the heterogeneity test are shown in Additional file: Supplementary Table S2. Therefore, we used a random-effects model to analyze the relationship between BT combined with HT and the 5-year bPFS rates of patients with prostate cancer. The summary RR for the relationship was 1.04 (95% CI: 1.01–1.08). This result indicated that BT combined with HT increased the 5-year bPFS rates of patients with prostate cancer compared with BT monotherapy. In order to find the source of the heterogeneity, we conducted a subgroup analysis in which the studies were organized into subgroups by geographic region (where they were conducted) or age of the patients. We found that BT combined with HT did not increase the 5-year bPFS rates of patients with prostate cancer in studies from Europe (RR = 1.04, 95% CI: 0.97–1.13, *P* = 0.201) and that the heterogeneity among these studies was high (Q = 13.13, *I*^2^ = 69.5%; *P* = 0.011, Tau^2^ = 0.0019). However, BT combined with HT increased the 5-year bPFS rates of patients with prostate cancer in studies from North America (RR = 1.05, 95% CI: 1.01–1.09, *P* = 0.007), but the heterogeneity among these studies was also high (Q = 13.02, *I*^2^ = 53.9%; *P* = 0.043, Tau^2^ = 0.0011) (Figure 3A). In the other subgroup analysis (Figure 3B), the heterogeneity among the indicated studies was low (Q = 0.49, *I*^2^ = 0.00%; *P* = 0.783, Tau^2^ = 0.0000) in the studies with a median age ≤65 years, but the heterogeneity among the indicated studies was high (Q = 17.94, *I*^2^ = 66.5%; *P* = 0.006, Tau^2^ = 0.0021) in the studies with a median age of 65–75 years. We conducted an interactive analysis and found that there were no significant differences between subgroups in different regions (*Z* = −0.78, *P* = 0.436) or in the different median age subgroups (*Z* = −01.32, *P* = 0.186). At the same time, we performed sensitivity analyses to verify the effect of each study on the overall estimate by omitting a study each time and determining the overall estimates for the remaining studies. The results of the sensitivity analysis showed good consistency and indicated that ignoring any one of the studies did not significantly affect the combined estimate; the range of the results was quite narrow. The results of the sensitivity analyses are shown in Figure 5. Our results indicated that the pooled estimate of our analysis was statistically robust.

**Figure 4:**
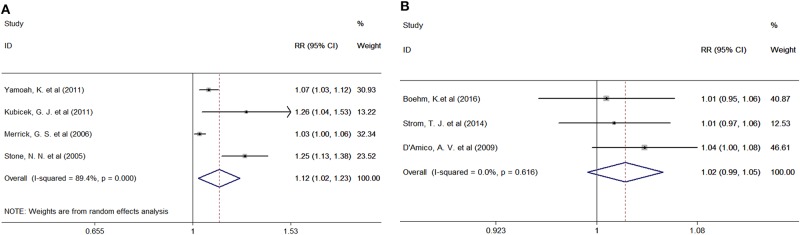
**(A)** Forest plots of the association between BT combined with HT and the 10-year bPFS rates of patients with prostate cancer. **(B)** Forest plots of the association between BT combined with HT and the 5-year OS rates of patients with prostate cancer. RR, Relative Risk; CI, Confidence Interval.

Four studies provided the number of patients in the experimental and control groups and their 10-year bPFS rates. A forest plot of the association between BH combined with HT and the 10-year bPFS rates of patients with prostate cancer is shown in Figure 4A. Our analysis revealed that these studies had high heterogeneity (Q = 28.26, *I*^2^ = 89.4%, *P* = 0.001, Tau^2^ = 0.0063), so we used a random-effects model. The summary RR for the relationship was 1.12 (95% CI: 1.02–1.23), indicating that BT combined with HT also increased the 10-year bPFS rates of patients with prostate cancer compared with BT monotherapy.

**Figure 5:**
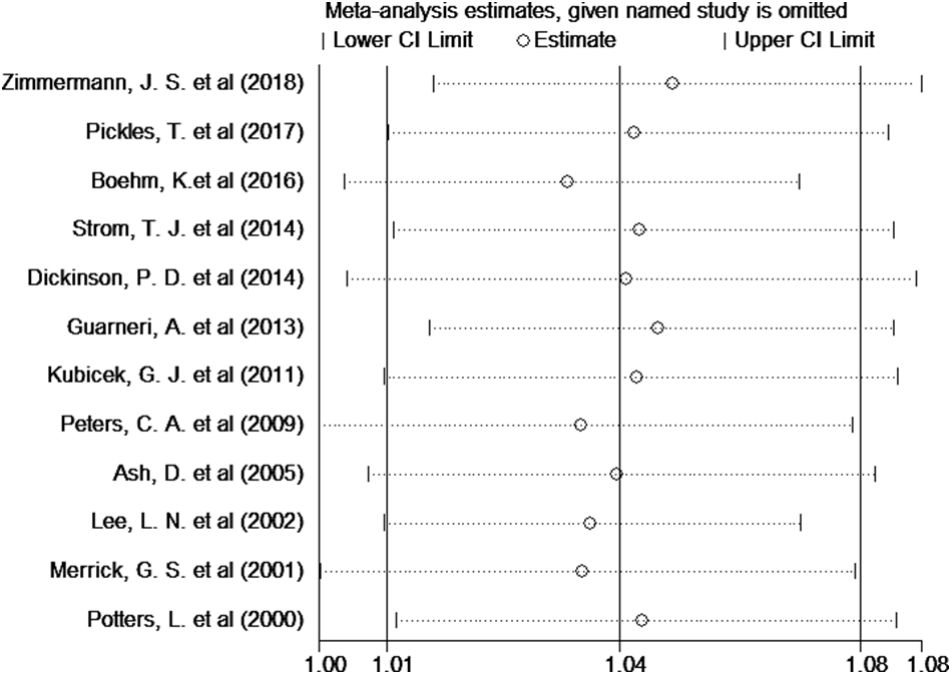
Sensitivity analysis of the 5-year bPFS rates of patients with prostate cancer.

### Overall Survival

Three studies provided the number of patients in the experimental and control groups and their 5-year OS rates. A forest plot of the association between BH combined with HT and the 5-year OS rates of patients with prostate cancer is shown in Figure 4B. Our analysis revealed that this study had little heterogeneity (Q = 0.97, *I*^2^ = 0.0%, *P* = 0.616, Tau^2^ = 0.0000), so we used a fixed-effects model. However, the summary RR for the relationship was 1.02 (95% CI: 0.99–1.10). This result indicated that BT combined with HT, compared with BT monotherapy, was positively associated with the 5-year OS rates of patients with prostate cancer, although the association was not significant.

### Publication Bias

**Figure 6:**
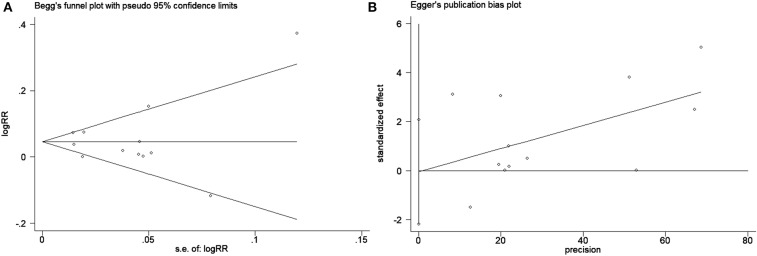
Publication bias test for evaluating the 5-year bPFS rates of patients with prostate cancer. **(A)** Begg's funnel plot with pseudo 95% confidence limits and **(B)** Egger's publication bias plot.

Publication bias was assessed using Begg's funnel plots and Egger's test (Figure 6). The results indicated that there was no publication bias for the analysis of the 5-year bPFS rates (Begg's test, *P* = 1.00; Egger's test, *P* = 0.963). As the number of studies included in the analyses of the 10-year bPFS rates and 5-year OS rates was small (<10), we did not construct a funnel plot. Because the test efficiency is low when the studies are too few, it is not enough to test if the funnel diagram is asymmetric (34).

## Discussion

Through a systematic review of the literature, 16 studies on BT combined with HT in the treatment of localized prostate cancer from North America and Europe were analyzed in a meta-analysis, which is the most comprehensive review of this topic at present.

BT includes short-term implant therapy and permanent-particle implant therapy, of which 125I or 103Pd implants are currently the most popular forms of implant therapy (33). After accurate positioning by the three-dimensional system, the radioactive source is sealed and placed directly into the prostate for implantation treatment. Prostate cancer brachytherapy was first performed by Barringer in 1915 (35, 36) and was adopted for the first time in the 1970s, when the retro-pubic method was widely used (37). Ultrasound-guided permanent prostatic implantation appeared in the early 1980s and has been used worldwide. Holm and his colleagues first described the ultrasound-guided transperineal technique in 1983 (38). Sylvester et al. reported the treatment of 215 cases of localized prostate cancer with 125I seed implantation alone. The follow-up period was 15 years, and the disease-free survival rate was 80.4% (39). Similarly, in a study with 5- and 10-year follow-ups, Martinez et al. reported OS rates of 94% and 84%, respectively, among 700 patients treated with 125I seed implantation (40). The report further confirmed the satisfactory long-term efficacy of BT for the treatment of prostate cancer. BT has also been used to treat breast cancer (41, 42), skin cancer (43), lung cancer (44, 45), head and neck tumors (46), esophageal cancer (47, 48), bile duct cancer (49), soft tissue sarcomas (50), and gynecological tumors (51). Furthermore, BT has been found to be a highly effective and safe treatment, providing a good alternative to surgical removal of the prostate, breast and cervix while reducing the risks of some long-term side effects (52).

HT is one of the main treatments for advanced or metastatic prostate cancer, and the role of HT in localized prostate cancer has received increasing attention in the research literature (53). Multiple prospective trials of men with localized prostate cancer have shown that RT combined with HT improves overall survival compared with RT alone (54–56). The rationale supporting the combination of HT and RT is based on an inference about patients with prostate cancer from a large number of randomized RT trials. EBRT combined with HT has been shown to improve survival through increased local control (57). This therapy is suitable for patients with intermediate-risk and high-risk prostate cancer (58). As stated in the Introduction, Lee et al. reported improved bPFS rates of patients with prostate cancer who received BT combined with HT (12). However, the findings of some studies are inconsistent with the above results. To address this controversy, we conducted a meta-analysis to clarify the efficacy of BT combined with HT, and our data showed that BT combined with HT increased the 5-year (RR = 1.04, 95% CI: 1.01–1.08) and 10-year (RR = 1.12, 95% CI: 1.02–1.23) bPFS rates of patients with prostate cancer compared with BT monotherapy. However, BT combined with HT, compared with BT monotherapy, did not increase the 5-year OS rates of patients with prostate cancer.

Although HT is well-tolerated by most patients, it is associated with some adverse medical sequelae. For example, HT has been reported to increase the risk of fractures (59), obesity, hyperlipidemia, diabetes and metabolic syndrome (60). However, in our meta-analysis, these events were not reported in the 16 articles we included, so we were unable to conduct a comparative analysis. Whether BT combined with HT has the potential to increase the incidence of these complications requires a large number of randomized controlled trials to confirm.

This meta-analysis has the following advantages. First, it seems to be the first meta-analysis to evaluate whether BT combined with HT can increase 5-year or 10-year bPFS rates and 5-year OS rates of patients with prostate cancer. The results showed that BT combined with HT increased the 5-year and 10-year bPFS rates but not the 5-year OS rates. Second, a sensitivity analysis showed that the results did not change significantly after removing any one of the studies from the meta-analysis, indicating that the results are very robust. Finally, the 16 studies included in the meta-analysis were from different countries (giving it good representativeness), with high NOS scores and strict adherence to our inclusion and exclusion criteria, indicating that the quality of the studies was high and the results were applicable to the general population. Therefore, the results of the meta-analysis are stable and reliable.

However, this study has several limitations. First, the number of included studies that provided 5-year OS rates or 10-year bPFS rates in the analysis was small (<10); therefore, we did not generate a funnel plot and were unable to detect publication bias. Second, we were unable to account for the influence of some important confounding factors that might have affected the results of our comprehensive analysis. Finally, the original studies did not provide data on treatment-related side effects, so comprehensive subgroup analyses were not performed. Although randomized trials provide the strongest evidence, there is not always reliable evidence in the field of oncology. Clinical practice is often based on observational studies, multiple small trials and even clinical experience alone (61). Therefore, a meta-analysis might be one of only a few available research methods for assessing the effectiveness and efficacy of clinical treatments.

In summary, BT combined with HT increased the bPFS rates of patients with localized prostate cancer, but the combination did not improve their OS rates. The results of this study provide new ideas for the treatment of localized prostate cancer in the future. As this study was a meta-analysis of retrospective cohort studies, further prospective randomized controlled studies are needed to reach reliable conclusions.

## Author Contributions

XZ, DJ, MD, and XH designed and conceived the research. ZL, YL, and XZ searched the database. XZ and JC analyzed the data. DJ, BH, JL, and XH wrote the draft. All authors reviewed the manuscript and approved the final manuscript.

### Conflict of Interest

The authors declare that the research was conducted in the absence of any commercial or financial relationships that could be construed as a potential conflict of interest.
