# Supplementary material for: Brachytherapy Combined With or Without Hormone Therapy for Localized Prostate Cancer: A Meta-Analysis and Systematic Review
Source: Front Oncol. 2020 Feb 19;10:169. doi: 10.3389/fonc.2020.00169 (PMC7042206; doi:10.3389/fonc.2020.00169)
Supplement: Supplementary file 1 [file Table_1.pdf]

Table S1. The detailed search strategy

Electronic databases	Search	Search strategy	Results
**Medline**	#1	"Prostate cancer"[Title/Abstract]	104667
	#2	"Prostate carcinoma"[Title/Abstract]	6453
	#3	"prostatic neoplasms"[Title/Abstract]	2090
	#4	(("prostatic neoplasms"[Title/Abstract]) OR "Prostate carcinoma"[Title/Abstract]) OR "Prostate cancer"[Title/Abstract]	109006
	#5	Brachytherapy [Title/Abstract]	16395
	#6	Hormone [Title/Abstract]	367674
	#7	Androgen [Title/Abstract] androgen	57857
	#8	(androgen[Title/Abstract]) OR hormone[Title/Abstract]	85294
	#9	#4 AND #5 AND #8	**765**
**Embase**	#1	'prostate cancer':ab,ti OR 'prostate carcinoma':ab,ti OR 'prostatic neoplasms': ab,ti	160257
	#2	'brachytherapy':ab,ti	27400
	#3	'hormone':ab,ti OR 'androgen':ab,ti	504572
	#4	#1 AND #2 AND #3	**1584**
**Web of Science**	#1	TOPIC: (Prostate cancer) OR TOPIC: (Prostate carcinoma) OR TOPIC: (prostatic neoplasms)	297672
	#2	TOPIC: (hormone) OR TOPIC: (androgen)	1128526
	#3	TOPIC: (brachytherapy)	38365
	#4	#3 AND #2 AND #1	**1971**
**Cochrane Library**	#1	(Prostate cancer):ti,ab,kw OR (Prostate carcinoma):ti,ab,kw OR (prostatic neoplasms):ti,ab,kw (Word variations have been searched)	10570
	#2	(hormone):ti,ab,kw OR (androgen):ti,ab,kw (Word variations have been searched)	43236
	#3	(brachytherapy):ti,ab,kw	1578
	#4	#1 and #2 and #3	**148**
