# Supplementary material for: Brachytherapy Combined With or Without Hormone Therapy for Localized Prostate Cancer: A Meta-Analysis and Systematic Review
Source: Front Oncol. 2020 Feb 19;10:169. doi: 10.3389/fonc.2020.00169 (PMC7042206; doi:10.3389/fonc.2020.00169)
Supplement: Supplementary file 2 [file Table_2.pdf]

Table S2. Tests of heterogeneity

5-year PFS	Q value	Degrees of freedom	P value	I^2	Tau^2
Europe	13.13	4	0.011	69.5%	0.0019
North America	13.02	6	0.043	53.3%	0.0011
NA	0.00	1	0.985	0.0%	0.0000
66-75	17.94	6	0.006	66.5%	0.0021
≤65	0.49	2	0.783	0.0%	0.0000
Overall	31.40	11	0.001	65.0%	0.0014
**5-year OS**	**Q value**	**Degrees of freedom**	**P value**	I^2	Tau^2
Overall	0.97	2	0.616	0.0%	0.0000
**10-year PFS**	**Q value**	**Degrees of freedom**	**P value**	I^2	Tau^2
Overall	28.26	3	0.000	89.4%	0.0063
